# Moderators and mediators of change of an internet-based mindfulness intervention for college students: secondary analysis from a randomized controlled trial

**DOI:** 10.3389/fdgth.2023.1179216

**Published:** 2023-06-27

**Authors:** Ann-Marie Küchler, Fanny Kählke, Leandra Bantleon, Yannik Terhorst, David Daniel Ebert, Harald Baumeister

**Affiliations:** ^1^ Department of Clinical Psychology and Psychotherapy, Institute of Psychology and Education, Ulm University, Ulm, Germany; ^2^ Department for Sport and Health Sciences, Professorship for Psychology & Digital Mental Health Care, Technical University of Munich, Munich, Germany

**Keywords:** college, university, mindfulness, internet- and mobile-based intervention, moderation, longitudinal mediation

## Abstract

**Background:**

Existing evidence suggests internet- and mobile-based interventions (IMIs) improve depressive symptoms in college students effectively. However, there is far less knowledge about the potential mechanisms of change of mindfulness-based IMIs, which could contribute to optimizing target groups and interventions. Hence, within this secondary analysis of data from a randomized controlled trial (RCT), potential moderators and mediators of the effectiveness of the IMI StudiCare Mindfulness were investigated.

**Methods:**

Moderation and mediation analyses were based on secondary data from a RCT that examined the effectiveness of the 7-module IMI StudiCare Mindfulness in a sample of college students (intervention group: *n* = 217; waitlist control group: *n* = 127). Assessments were collected before (t0; baseline), 4 weeks after (t1; during intervention), and 8 weeks after (t2; post-intervention) randomization. Longitudinal mediation analyses using structural equation modeling were employed, with depressive symptom severity as the dependent variable. For moderation analyses, bilinear interaction models were calculated with depressive symptom severity and mindfulness at t2 as dependent variables. All data-analyses were performed on an intention-to-treat basis.

**Results:**

Mediation analyses showed a significant, full mediation of the intervention effect on depressive symptom severity through mindfulness (indirect effect, a*b = 0.153, *p* < 0.01). Only the number of semesters (interaction: *β* = 0.24, *p* = 0.035) was found to moderate the intervention's effectiveness on depressive symptom severity at t2, and only baseline mindfulness (interaction: *β* = −0.20, *p* = 0.047) and baseline self-efficacy (interaction: *β* = −0.27, *p* = 0.012) were found to be significant moderators of the intervention effect on mindfulness at t2.

**Conclusion:**

Our results suggest a mediating role of mindfulness. Moderation analyses demonstrated that the intervention improved depressive symptom severity and mindfulness independent of most examined baseline characteristics. Future confirmatory trials will need to support these findings.

**Clinical Trial Registration:**

The trial was registered a priori at the WHO International Clinical Trials Registry Platform via the German Clinical Studies Trial Register (TRN: DRKS00014774; registration date: 18 May 2018).

## 1. Introduction

Numerous studies have shown that college students are at risk of developing mental health problems (1–4). Given the multiple stressors young people face persuing their academic careers (5), it is not surprising that about one-third of them develop psychological disorders such as depression (21%) (6). During the COVID-19 pandemic college students have been confronted with additional challenges, resulting in an increase of mental health problems. For example, the prevalence of depression was estimated to be between 34% and 39% (7–9). On the other hand, utilization of support services has been reported to be as low as 20% (10–12) due to barriers such as the preference to handle problems alone, embarrassment, and the lack of professional help offers. Internet- and mobile-based interventions (IMIs) have been suggested as a potential solution to this problem (13, 14). IMIs can be used anonymously at a time and place suitable to the user and are scalable, enabling the provision of mental health services to a large number of students (15). Evidence suggests that mindfulness-based interventions, such as Mindfulness-Based Stress Reduction (MBSR) (16) and Acceptance and Commitment Therapy (ACT) (17), can effectively reduce mental health problems in college students (18) and can be successfully delivered via IMIs (19).

Existing research demonstrates that IMIs can be as effective as therapeutically guided face-to-face therapy (20). Recent meta-analytic results show mindfulness-based IMIs effectively improve mental health outcomes such as depressive symptoms (*g* = 0.34, 95% CI 0.18–0.50) (19). However, knowledge about how and for whom these interventions work is far less extensive (21, 22). Both the mechanisms of change, through which they work (mediators) and the circumstances, under which psychological interventions are effective (moderators), should be studied as they provide information for optimizing interventions and better adapting them to target groups (23, 24). Knowledge about mediators can identify the most effective treatment components, whereas knowledge about moderators can support tailoring interventions to specific subpopulations (23). Therefore, the current study aimed to identify potential moderators and mediators of change by analyzing secondary data from a randomized controlled trial (RCT) evaluating the effectiveness of the mindfulness-based IMI StudiCare Mindfulness in a college student population (*N* = 387) (25), as described *a priori* in the study protocol (26). The intervention consists of 7 modules based on elements of MBSR and ACT. Two different versions of StudiCare Mindfulness, an unguided one (UG) and a minimally guided one (“guidance on demand”, GoD), were demonstrated to be effective compared to a waitlist control group (WLC). Analyses revealed moderate to large effects on the primary outcome mindfulness after 4 weeks (*d* = 0.75–0.76, 95% CI 0.05–1.02), 8 weeks (*d* = 0.91–1.06, 95% CI 0.66–1.32), and 6 months (*d* = 0.87–1.03, 95% CI 0.61–1.29), as well as small effects on the secondary outcome depression after 4 weeks (*d* = −0.25–−0.22, 95% CI −0.49–0.02), 8 weeks (*d* = −0.44–−0.33, 95% CI −0.68–−0.08), and 6 months (*d* = −0.39–−0.31, 95% CI −0.64–−0.07).

Potential moderators and mediators to be investigated within RCTs should be carefully selected based on a strong theoretical foundation (23). When examining mechanisms in mindfulness-based IMIs, both variables specific to the theoretical framework that the mindfulness intervention is built upon (specific factors) and to the IMI setting in general (common factors associated with digital delivery) should be considered (27).

First, the development of mindfulness skills, resulting in a non-judgmental and non-reactive acceptance of all experiences, is a central goal of MBSR and is assumed to be a key mechanism leading to the improvement of mental health outcomes (16). In their systematic review and meta-analysis investigating mediators of change of MBSR and Mindfulness-Based Cognitive Therapy (MBCT) (28), Gu and colleagues found moderate, consistent evidence for mindfulness as a mediator of the intervention's effectiveness on mental health outcomes such as depression (21). However, the majority of the included studies were unable to investigate the temporal precedence of changes in mindfulness, which is a crucial criterion for the establishment of mediation (23) and can only be tested with longitudinal designs with at least three assessments. It is met if the intervention initially leads to a change in the mediator, which then leads to a change in the outcome variable. Hence, such studies are needed to confirm mindfulness as a mechanism of change.

Second, as StudiCare Mindfulness integrates elements of ACT (29), another potential mediator is cognitive defusion (CD). It refers to the ability to perceive cognitions and emotions as objective events occurring in our minds rather than to identify with these experiences (29). Thus, CD alleviates psychological well-being by distancing oneself from negative internal events. A systematic review confirmed the mediating role of CD on the effectiveness of ACT and established temporal precedence (22). However, only four studies were included in the analysis. Additionally, to our knowledge, CD as a mediator in the IMI setting has only been studied and confirmed in two RCTs by one research group (30, 31). Thus, results need to be replicated across studies, samples, conditions, and settings to establish mediation (23).

Third, theoretical considerations (32) and existing evidence (33, 34) have suggested emotion regulation (ER) as a potential mediator of mindfulness-based interventions. ER can be defined as the process of influencing emotions in terms of when and how a person experiences and expresses emotions (35). Two such strategies are cognitive reappraisal, an antecedent-focused, experience-modifying strategy, and expressive suppression, a response-focused, expression-modifying strategy (36). Reappraisal has been hypothesized to be a process that occurs during mindful meditation practice through reinterpreting stressful events as helpful or meaningful (32). For example, a large survey study (*N* = 1,151) found reappraisal and supression to mediate the relationship between mindfulness and depression (37). A RCT found that changes in ER difficulties mediate the relationship between mindfulness and psychological distress in participants of a mindfulness-based IMI (38). This was replicated in another study that found partial mediation of the effectiveness of a mindfulness-based IMI on mental health improvement by ER (39). However, evidence is still scarce, and more research is needed to confirm these results.

Fourth, mindfulness interventions might work by enhancing clarity about one's internal experiences, which might contribute to managing negative emotions (40). An essential construct in this context is alexithymia, a trait that entails difficulties identifying and communicating emotions (41). Alexithymia is associated with various mental health problems and its reduction has been shown to be beneficial to physical and mental health (42). A systematic review examining the effect of mindfulness-based interventions on the outcome of alexithymia (4 RCTs) found significant improvement compared to active and passive control groups (42). Additionally, evidence suggests that improvements in emotional clarity mediate the relationship between mindfulness and mental health (40). However, to our knowledge, no study has tested a potential mediating role of alexithymia reduction on mental health outcomes in mindfulness-based interventions.

Fifth, self-efficacy (SE) has been proposed as a potential mediator of IMI effectiveness (43). Perceived SE refers to a person's belief that they can successfully perform a behavior to produce a specific outcome (44). Thus, SE will influence the initiation and persistence of coping efforts, e.g., whether and how someone will perform particular stress management strategies. Because it is in the nature of self-help interventions to empower participants to help themselves, SE could be a specifically relevant mediator in the context of IMIs. Evidence suggests SE as a mediator of face-to-face psychotherapy (45, 46), but it has not been systematically studied in the IMI setting. To our knowledge, only one study has examined and confirmed the mediating role of mental health SE on mental health outcomes (47). Another study found the closely related construct of “perceived control” to mediate the effects of a CBT-based IMI on depressive symptoms (48). So far, no trial has studied perceived SE in mindfulness-based IMI.

To investigate the potential differential effectiveness of mindfulness-based IMI, moderation analyses should be conducted. To date, different moderators of change have been studied in mindfulness-based interventions, e.g., sociodemographic variables (49), pre-treatment mindfulness (50, 51), and baseline symptomatology (49, 52). Additional moderators of IMI effectiveness that can be derived from other treatment approaches are expectations regarding the intervention's effectiveness (53) and pre-treatment SE (47). Overall, the evidence regarding moderators of effectiveness in mindfulness-based IMIs is scarce, inconclusive, and often exhibits methodological limitations (e.g., insufficient power). Therefore, further research is urgently needed.

In summary, intervention research should routinely include exploratory analyses of moderators and mediators of change (24). However, with regard to mindfulness-based IMIs, such research is still in its infancy (49), warranting further investigation as such factors might be specific to therapeutic approaches (54) or settings (27). Therefore, in the presented secondary analysis of data gathered in a RCT evaluating the effectiveness of the IMI StudiCare Mindfulness, we investigated (1) the potential moderating effect of various variables on the intervention's effectiveness, and (2) several potential mediators as outlined above, considering three assessment time points, thus enabling the establishment of temporal precedence. An overview of the examined moderator and mediator variables can be found in Table 1.

**Table 1:** Overview of potential moderator and mediator variables examined in this study.

Mediator variables	Moderator variables
Mindfulness	Sociodemographic characteristics
Cognitive defusion	Study-related characteristics
Emotion regulation: cognitive reappraisal	Baseline mindfulness
Emotion regulation: expressive suppression	Baseline depressive symptom severity
Clarity about one's internal experiences (i.e., alexithymia)	Baseline self-efficacy
Perceived self-efficacy	Expectations and credibility
	Previous help-seeking
	Use of alternative support offers during the participation

The dependent variable was depressive symptom severity at t2 for the mediation analyses and mindfulness and depressive symptom severity at t2 for the moderation analyses.

## 2. Methods

### 2.1. Study design

**Figure 1:**
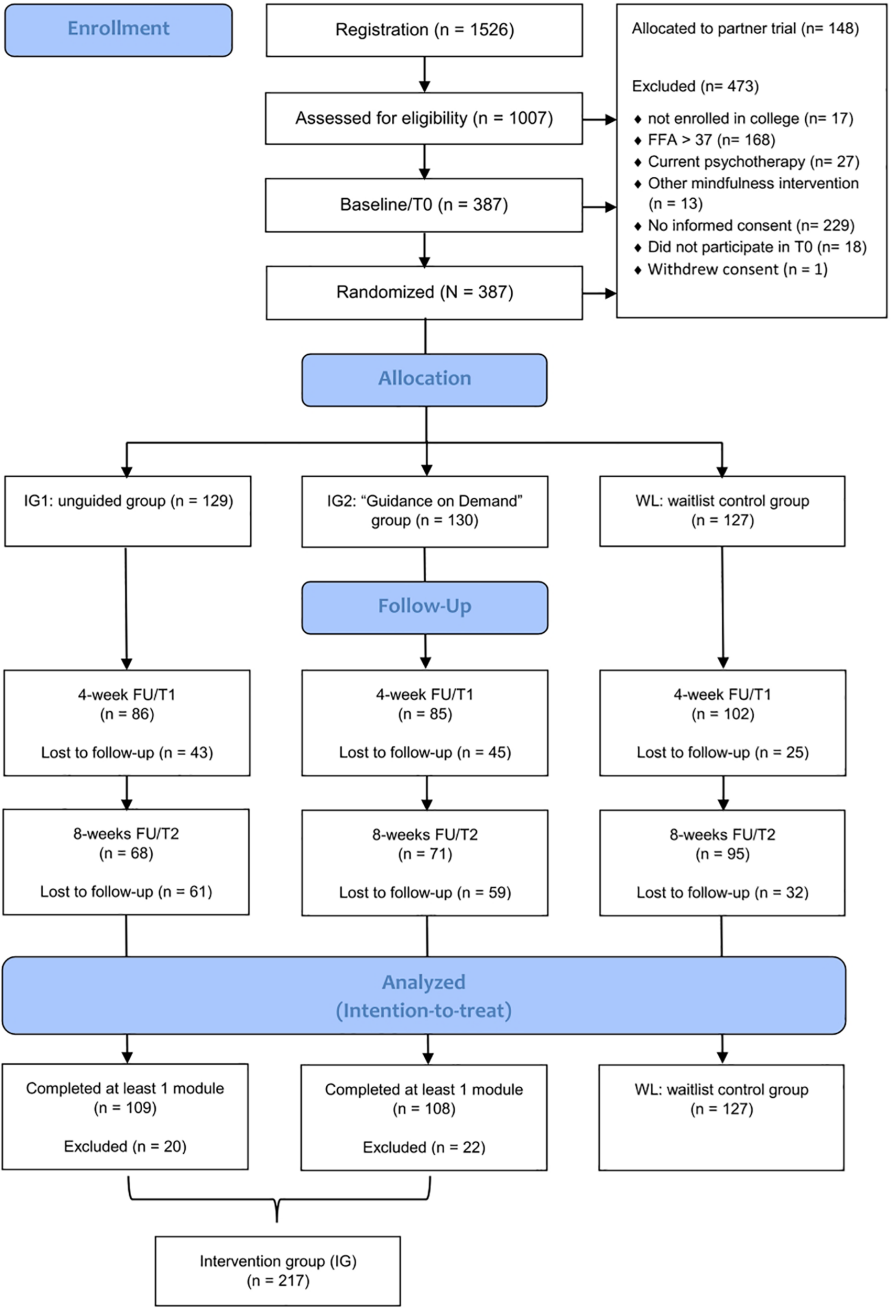
Flow diagram (see Küchler et al. (25); modified).

This secondary, time-lagged mediation and moderation study was based on a three-armed randomized controlled trial (RCT), which was originally designed to evaluate the effectiveness of two versions of the Internet- and mobile-based intervention (IMI) StudiCare Mindfulness in comparison to a waitlist control group (WL) as well as to each other (see Figure 1 for flowchart). One intervention group (UG) received no additional guidance (unguided) to the intervention, the other (GoD) received additional so-called “guidance on demand”, a form of guidance where participants can ask their e-coach (a trained psychologist) for support whenever they need it. For the scope of this secondary study, the two intervention groups were combined (see Section 2.9). Additionally, all groups had unrestricted access to usual treatment options (TAU). The trial was conducted within the StudiCare project (www.studicare.com), which investigates and promotes college student mental health by providing IMIs for various psychological and behavioral subjects. The trial was conducted according to the CONSORT 2010 (55) statement and registered *a priori* at the WHO International Clinical Trials Registry Platform via the German Clinical Studies Trial Register (TRN: DRKS00014774; registration date: 05/18/2018). Details on the study design (26) of the main study can be obtained from the study protocol and the publication on the main analysis (25).

### 2.2. Eligibility criteria

Written informed consent was obligatory to participate in the trial. Further, participants had to meet the following inclusion criteria: (a) aged 18 or older, (b) enrolled in university or college, (c) sufficient German language skills, (d) internet access, (e) moderate to low mindfulness (Freiburg Mindfulness Inventory FMI < 37, corresponding to a FMI mean based on the general population (56). Participants were excluded if they currently underwent psychotherapy or any kind of mindfulness intervention at the time of the screening. To be included in the secondary moderation and mediation analyses, participants additionally had to (f) have completed a minimal intervention dose of at least one module post-intervention (t2; 8 weeks after randomization).

### 2.3. Setting/recruitment

Participants were recruited at 18 cooperating colleges in Germany, Austria and Switzerland via circular e-mails, flyers and posters, social media, student unions, and student counseling. All recruitment channels led to the StudiCare homepage, where students found detailed information about StudiCare Mindfulness and registration. If students successfully completed an online screening, they were either assigned to a partner trial (students of Ulm University) (57) or the current trial (students of all other colleges). After the provision of informed consent, they completed the baseline assessment and were then randomized into one of the three groups. UG and GoD participants received immediate access to StudiCare Mindfulness, whereas WL participants received access after completing the follow-up assessment (6 months after randomization).

### 2.4. Randomization

Randomized allocation was conducted by an independent researcher not otherwise involved in the study. Permuted block randomization was performed using an automated, online-based randomization program (58) with an allocation ratio of 1:1:1 and randomly arranged variable block sizes of 6, 9 and 12.

### 2.5. Intervention

StudiCare Mindfulness comprises seven core modules and two booster sessions (4 and 12 weeks after the last core module). Modules were unlocked sequentially and weekly module completion was recommended, with each module taking about 40–60 min to work through. Module content is based on elements of Acceptance and Commitment Therapy (ACT) (17), Mindfulness-Based Stress Reduction (MBSR) (16) and general stress management techniques (59), and is diversely and interactively designed (e.g., including psychoeducational texts, case-examples, self-reflection exercises, meditation audio files). As homework assignments, participants are practicing mindfulness exercises between modules, which are reflected at the beginning of each module. For a detailed description of the intervention, see Table 2 as well as the study protocol (26). To participants of the GoD group, e-coach guidance was provided via the Minddistrict platform's message function whenever they actively requested it. E-coaches were supervised psychologists and gave semi-standardized feedbacks to participants' module input that was focused on motivation and encouragement. In contrast, UG participants only received short standardized messages that were automatically sent by the platform after completion of each module. Both groups were sent automatic reminders if they had not logged in for more than 7 days. Additionally, they could sign up for an SMS-coach to receive motivational text messages every other day. Participants could access StudiCare Mindfulness via the Minddistrict platform (www.minddistrict.com, accessed 18 February 2023) on a 24/7 basis using a personal username and password. All transferred data was secured based on ISO27001 and guidelines NEN7510.

**Table 2:** Intervention content [see (25)].

Module	Aims and content	Examples of exercises and assignments
1. Being in the here and now	Introducing the concept of mindfulness	Reviewing most and least mindful moments of the day; “body scan” meditation; taking mindful walk
2. Mindful body perception	Practicing awareness of body signals	Testing one's heartbeat perception; practicing “heart meditation”; mindful eating and drinking
3. A new perspective on stress	Distancing oneself from stress-inducing thoughts	Identifying former ways of coping with stress; learning techniques to challenge automatic thoughts; “mindful perception of body posture” meditation
4. Developing beneficial thoughts	Getting to know alternative ways of thinking	Identifying one's “stress patterns” and developing and internalizing beneficial thoughts; “mindful breathing” meditation
5. What makes your life valuable?	Identifying one's values and pursuing one's goals	Writing a speech for one's 70th birthday; setting and pursuing goals with the SMART technique; variation of “body scan” meditation
6. Being mindful towards yourself	Learning how to appreciatively accept one's personality traits	Exercise to identify different personality traits and corresponding automatic reactions; learning to accept and appreciate all personality traits; “loving kindness” meditation
7. Training your body and senses	Exercising the ability to enjoy and getting acquainted with the practice of yoga	Mindful chocolate eating exercise; mindful yoga exercises
Booster 1 (4 weeks after completion of module 7)	Repeating module 1–3 and mindfulness exercises	Choosing favorite mindfulness exercises; setting goals for their implementation in the coming weeks
Booster 2 (12 weeks after completion of module 7)	Repeating modules 4–7 and ensuring long-term integration of mindfulness into daily life	Reviewing pursuit of goals in the last two months; identifying potential barriers and developing solutions

### 2.6. Control condition

At the beginning of the study, WL participants received a leaflet about alternative support options (such as helplines, university counselling services) at the beginning of the study and had unrestricted access to usual treatment options (TAU) throughout their participation. They were able to access an unguided version of StudiCare Mindfulness six months after randomization.

### 2.7. Assessments and outcomes

Assessments were conducted via the online survey platform “Unipark” (www.unipark.com, accessed 18 February 2023) before (t0; baseline), 4 weeks (t1; intermediate), and 8 weeks (t2; post-treatment) after randomization (see Figure 1) to enable establishment of temporal precedence. Here, only a subset of instruments that are relevant to the proposed mediation and moderation analyses is presented. For an overview of all outcomes of the RCT, see Küchler et al. (26).

#### 2.7.1. Dependent variable

##### 2.7.1.1. Depressive symptom severity

Depressive symptom severity was selected as dependent variable in accordance with the majority of the existing mediation studies on mindfulness-based interventions (21). It was assessed with the depression module of the Patient Health Questionnaire (PHQ-9) (60). Within the PHQ-9, 9 items are rated on a 4-point scale (0 = “not at all” to 3 = “nearly every day”). The PHQ-9 is a widely used depression screening instrument and was shown to have good diagnostic properties and a high internal consistency of *α* = 0.89 (61).

#### 2.7.2. Mediator variables

##### 2.7.2.1. Mindfulness

Mindfulness was assessed via the 14-item short scale of the Freiburg Mindfulness Inventory (FMI) (56), which used a 4-point scale ranging from 1 = “rarely” to 4 = “almost always”. The FMI has demonstrated high internal consistency (*α* = 0.84) (62) and sensitivity to change (56) in previous studies.

##### 2.7.2.2. Self-efficacy

To measure perceived general self-efficacy, the Self-efficacy Scale (SES) (63) was employed. 10 items are rated on a 4-point scale ranging from 1 = “not at all true” to 4 = “very true”. The SES has demonstrated good internal consistency of 0.75–0.91 in previous research (64).

##### 2.7.2.3. Cognitive fusion

Cognitive fusion was assessed with the 7-item Cognitive Fusion Questionnaire (CFQ-D) (65) on a 7-point scale ranging from 1 = “never true” to 7 = “always true”. Previous research has found internal consistency to be high with a Cronbach's *α* = 0.95 (65).

##### 2.7.2.4. Emotion regulation

Habitual use of the two emotion regulation strategies “cognitive reappraisal” (6 items) and “expressive suppression” (4 items) was assessed with the Emotion Regulation Questionnaire (ERQ) (36) on a 7-point scale ranging from 1 = “strongly disagree” to 7 = “strongly agree”. The ERQ has previously demonstrated good internal consistency (reappraisal: *α* = 0.74; suppression: *α* = 0.76) (66).

##### 2.7.2.5. Alexithymia

Using the Toronto Alexithymia Scale (TAS-20) (41, 67), alexithymia was assessed with 20 items which are rated on a five-point scale (1 = “strongly disagree”; 5 = “strongly agree”). The TAS-20 has three subscales: “difficulty identifying feelings” (DIF), “difficulty describing feelings” (DDF) and “externally oriented thinking” (EOF) and was found to have good internal consistency (*α* = 0.85–0.86) (68).

#### 2.7.3. Moderator variables

Several variables were assessed that potentially moderate the dependent variables mindfulness (FMI) and depressive symptom severity (PHQ-9) at t2: age, gender, nationality, marital status, and number of semesters, previous experience with mindfulness (assessed retrospectively at t2), previous help-seeking (mindfulness and psychotherapy), and use of alternative support services. In addition, participants rated the six items of the Credibility Expectancy Questionnaire (CEQ) (69). The CEQ consists of three items for the credibility subscale (“how believable, convincing, and logical the treatment is” and three for the expectancy subscale (“improvements that clients believe will be achieved” (70), each measured on a 9-point Likert Scale to assess positive expectations and credibility of StudiCare Mindfulness. The CEQ's internal consistency was found to be high in previous research, Cronbach's *α* = 0.84–0.85 (69). Finally, mindfulness (FMI), depression (PHQ-9), and self-efficacy (SES) at baseline (t0) were examined as potential moderators.

### 2.8. Statistical analyses

#### 2.8.1. Mediation analysis

Mediation effects for each potential mediator were investigated in separate structural equation models (SEM), following the principles of time-lagged mediation analyses by Cole and Maxwell (71). For the independent variable “group”, the two intervention groups (UG and GoD) were combined (1 = intervention group, IG) and compared to the waitlist control group (0 = WLC). This decision was based on findings of the effectiveness analyses (72), which revealed no substantial differences in depressive symptom severity and pre-defined moderator and mediator variables between UG and GoD at all assessment times. Additionally, guidance utilization in the GoD group was very low (15%), resulting in high comparability of the intervention actually received by both groups. Depression severity (PHQ-9) was set as dependent variable. Full information maximum likelihood (FIML) was applied to estimate model parameters, as this approach allows for accurate estimation even in the presence of missing data (73). Model fit was evaluated with root mean square error of approximation (RMSEA) and standardized root mean square residual (SRMR) as the *χ*^2^-test has a tendency to reject mis-specified models too sensitively (74–76). Standard modeling criteria were used to establish cut-off values for an acceptable goodness of fit: RMSEA < 0.06, SRMR < 0.08 (77). Nested models were compared via model deviance tests using *χ*^2^-tests.

Following Cole and Maxwell (71), we conducted five steps for testing mediational effects with longitudinal data using SEM. These steps aimed to find the most parsimonious model with a good fit to the data (71). First, the measurement model (“full model”) was tested allowing “group” and all latent variables to correlate with each other. This model constituted the basis for all model comparisons in the following steps. Second, the equality of various parameters across waves (i.e., assessment times) was evaluated by testing for equilibrium and factorial invariance. Third, it was tested whether variables not included in the model may account for the covariation between latent variables. Fourth, we investigated which additional causal paths should be added to the longitudinal full mediation model. Finally, in the fifth step, direct effects (c'), indirect effects (a*b) and total effects (c = c' + a × b) were estimated using the most parsimonious model with a good model fit derived from the previous steps. Autoregressive effects between waves were set equal to substantiate the stationarity assumption. As the direct effect (c'-path) was only significant in the SEM of one potential mediator (expressive suppression), mediation analyses for the other four mediator variables were also calculated with SEM that did not include the c'-path as a sensitivity analysis.

#### 2.8.2. Moderation analysis

For the moderation analyses, dichotomous variables were dummy-coded, multicategorical variables were dichotomized (e.g., civil status into “single” vs. “in partnership”), and continuous variables were z-standardized. Bilinear interaction models were employed, with depression at t2 (post-intervention) serving as the dependent variable. Additionally, the same analyses were conducted with mindfulness at t2 as dependent variable, as it was the primary outcome variable in the main study and this was specified in the study protocol (26). In both cases, “group” was set as independent variable. If significant interactions between group and moderator were detected, moderation effects were subsequently examined using simple slopes analyses. This involved testing high (+1 SD), average (M), and low (−1 SD) levels of the moderation variable.

All analyses were performed using the software R (78). The R package “lavaan” (version 0.6–8) (79) was used for SEM. All statistical analyses were conducted following the intention-to-treat (ITT) principle. Missing data was presumed to be at random (73). To account for this, the full information maximum likelihood estimator was utilized in the mediation analyses to handle missing values. For the moderation analysis, ITT was operationalized via multiple imputation using chained equations (80). Predictive mean matching (81) was employed to impute *N* = 20 datasets. ITT analyses were conducted for each imputed dataset, and results were subsequently pooled according to Rubin's rule (82). Only participants who completed a minimal dose of treatment (at least one module of StudiCare Mindfulness) were included in the analyses (*N* = 344).

## 3. Results

### 3.1. Participants

Of the 1,526 college students that registered for the trial, 405 fulfilled eligibility criteria and 386 were included and randomized into the three study groups (see Figure 1 for flowchart). The resulting sample sizes were *n* = 129 for the unguided group (UG), *n* = 130 for the “guidance on demand” group (GoD), and *n* = 127 for the waiting list control group (WL). After excluding participants that had not received a minimal dose of at least one module of StudiCare Mindfulness and combining the two intervention groups (IG), this resulted in *n* = 217 (IG) and *n* = 127 (WL). Participants were mainly female (74%) and on average 25.7 years old (*SD* = 5.28) (see Table 3).

**Table 3:** Baseline characteristics.

	All (*N* = 344)		IG (*n* = 217)		WL (*n* = 127)	
	*n*	%	*n*	%	*n*	%
Sociodemographic characteristics						
Age (M, SD)	25.65	5.28	25.49	5.02	25.87	5.70
Female Gender	163	74.4	162	74.7	95	74.8
Single	227	66.0	148	68.2	79	62.2
German Citizenship	275	79.9	170	78.3	105	82.7
Study characteristics						
Full-time student	280	81.4	179	82.5	101	79.5
Number of total semesters (M, SD)	9.00	5.32	8.91	5.13	9.14	5.66
Previous help seeking						
Psychotherapy experience	82	23.8	44	20.3	38	29.9
Mindfulness experience	130	37.6	89	41.0	43	33.8
PHQ-9 (M, SD)	9.28	4.38	9.33	4.33	9.17	4.47
FMI (M, SD)	29.82	4.78	29.88	4.83	29.71	4.71
SES (M, SD)	25.91	4.49	26.01	4.53	25.74	4.43
CEQ (M, SD)	38.82	7.25	38.89	7.20	38.80	7.35
Alternative support (t2, post-intervention)	105	30.3	66	29.5	41	32.3

CEQ, credibility/expectancy questionnaire; M, mean; N/n, number; PHQ-9, patient health questionnaire; SD, standard deviation.

### 3.2. Mediation analysis

**Figure 2:**
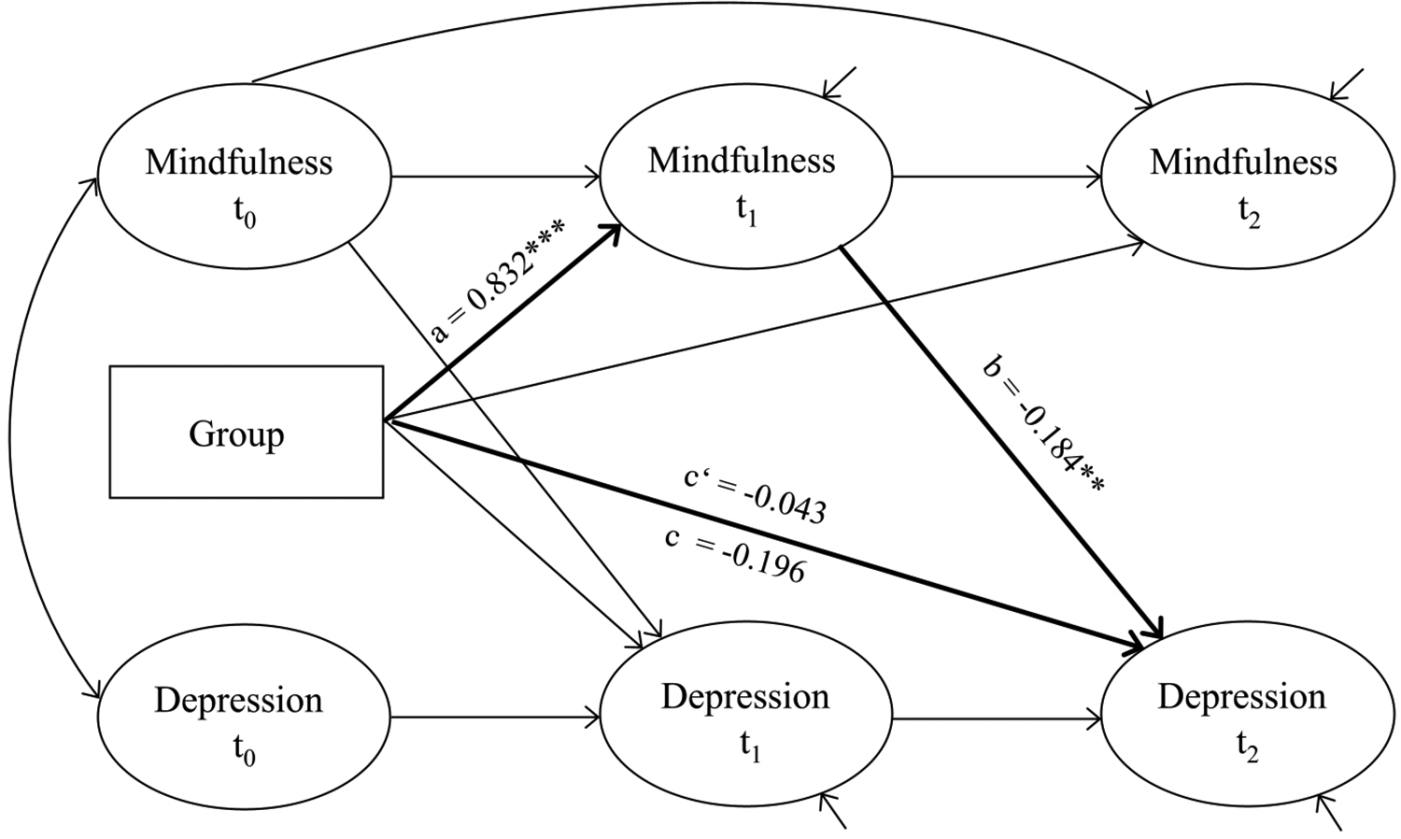
Path model for the mediator mindfulness. Ellipses are latent variables, rectangles are manifest variables. *p < 0.05, **p < 0.01, ***p < 0.001.

Tests of model fit and model comparisons within Cole and Maxwell's (71) steps 1–3 (described above) revealed a good fit of the measurement models and the presence of equilibrium and factorial invariance. This was modeled accordingly in all six final SEM. Factor loadings were set equal across waves and the residuals of the endogenous latent variables were allowed to correlate. Autoregressive effects between waves were set equal. Additional causal paths that were found to be significant in step 4 were included in the final SEM. Model fits of the final SEM of each mediator were good overall (RMSEA: 0.038–0.046; SRMR: 0.066–0.083) and can be obtained from Table 4. The only significant mediation was found for mindfulness (see Table 4 and Figure 2), whereas no other potential mediator variable demonstrated a mediating effect (see Table 2 and Supplementary Figures S1–S5 in the Supplementary Material for the respective results and path models). For mindfulness, we found a significant a-path (effect of StudiCare Mindfulness on mindfulness: *β* = 0.832, *p* < 0.001), a significant b-path (effect of mindfulness on depressive symptom severity: *β* = −0.184, *p* = 0.005), and a significant a*b-path (indirect effect of StudiCare Mindfulness on depressive symptom severity mediated through mindfulness: *β* = −0.153, *p* = 0.007). Both the c-path (total effect of StudiCare Mindfulness on depressive symptom severity: *β* = −0.196, *p* = 0.055) and c'-path (direct effect of StudiCare Mindfulness on depressive symptom severity: *β* = −0.043, *p* = 0.709) did not reach significance, implying full mediation of the effect of StudiCare Mindfulness on depressive symptom severity at t2 (post-intervention) through mindfulness at t1 (intermediate assessment). Substantiating the causal direction of the mediation effect, all backwards effects tested within step 4 did not reach significance, nor did inclusion of these paths significantly improve model fit. These included paths from depressive symptom severity at t0 to mindfulness at t1 (*β* = 0.041, *p* = 0.497), from depressive symptom severity at t1 to mindfulness at t2 (*β* = −0.007, *p* = 0.915), from depressive symptom severity at t0 to mindfulness at t2 (*β* = 0.092, *p* = 0.125) and from mindfulness at t0 to depressive symptom severity t2 (*β* = −0.039, *p* = 0.600). Sensitivity analyses with SEM excluding the c'-paths (direct effect) yielded comparable results (see Supplementary Table S1).

**Table 4:** Results of a longitudinal mediation analysis with three measurement time points.

Mediator	a-path	b-path	Direct effect c’-path	Indirect effect a × b	Total effect c-path = (a × b) + c'	RMSEA	SRMR
Mindfulness	0.832***	−0.184**	−0.043	−0.153**	−0.196	0.042	0.077
Self-efficacy	0.523***	−0.092	−0.154	−0.048	−0.202	0.041	0.073
Cognitive fusion	−0.337***	0.132*	−0.191	−0.044	−0.235*	0.042	0.066
ER—CR	0.404***	−0.100	−0.175	−0.040	−0.215*	0.044	0.071
ER—ES	−0.172	0.063	−0.211*	−0.011	−0.221*	0.038	0.070
Alexithymia	−0.229**	0.085	−0.200	−0.019	−0.220*	0.046	0.083

The outcome was PHQ-9 depressive symptom severity for each mediator. Coefficients are standardized. ER-CR, emotion regulation cognitive reappraisal; ER-ES, emotion regulation expressive suppression.

* *p* < 0.05.

** *p* < 0.01.

*** *p* < 0.001.

### 3.3. Moderation analysis

See Supplementary Tables S2, S3 for the detailed results of the moderation analyses. Regarding the dependent variable depressive symptom severity at t2, only “number of semesters” was found to be a significant moderator of the intervention's effectiveness (interaction: *β* = 0.24, 95% CI: 0.02–0.47, *p* = 0.035). Simple slope analyses revealed that the intervention's effectiveness on depressive symptom severity at t2 was most pronounced for IG participants with a low number of semesters (−1 SD; *β* = −0.65, 95% CI: −0.95–−0.18), compared to WL. Individuals with a high number of semesters (+1 SD; *β* = −0.18, 95% CI: −0.47–0.17) experienced the smallest benefits, with depressive symptom severity no longer being significantly reduced compared to WL. For the dependent variable mindfulness at t2, two variables were found to significantly moderate the intervention's effectiveness, baseline mindfulness (interaction: *β* = −0.20, 95% CI: −0.41–−0.00; *p* = 0.047) and baseline self-efficacy (SE) (interaction: *β* = −0.27, 95% CI: −0.48–−0.06; *p* = 0.012) compared to WL. In both cases, the intervention was more effective for participants with low baseline levels of these variables (baseline mindfulness: −1 SD; *β* = 1.15, 95% CI: 0.91–1.40; baseline SE: −1 SD; *β* = 1.21, 95% CI: 0.95–1.46) and less effective for participants with high baseline levels (baseline mindfulness: +1 SD; *β* = 0.75, 95% CI: 0.50–0.99; baseline SE: +1 SD; *β* = 0.70, 95% CI: 0.41–0.93).

## 4. Discussion

Within this study, we analyzed secondary data from a randomized controlled trial (RCT) evaluating the Internet- and mobile-based mindfulness intervention (IMI) for college students, StudiCare Mindfulness, to gain insights into potential moderating and mediating variables of the intervention's effectiveness. Mindfulness was found to fully mediate the intervention's effectiveness on depressive symptoms in the sense that the more mindfulness increased during the intervention, the more depressive symptoms decreased by the end of the intervention. All other examined mediators, i.e., self-efficacy, cognitive defusion, emotion regulation, and alexithymia, could not be confirmed as mediators. Only one of the examined variables (i.e., number of semesters) moderated the intervention's effectiveness on depressive symptom severity, whereas two variables (i.e., baseline mindfulness, baseline SE) were found to moderate the intervention effect on mindfulness.

### 4.1. Mediation analysis

The finding that mindfulness mediated the intervention's effectiveness on depressive symptom severity is in line with existing evidence. In a previous RCT evaluating a guided version of StudiCare Mindfulness, mindfulness was found to mediate improvements in depressive symptoms in a cross-sectional mediation analysis (72). Additionally, in their systematic review and meta-analysis, Gu and colleagues (21) found consistent moderate evidence for mindfulness as a mediator of Mindfulness-Based Stress Reduction (MBSR) (16) and Mindfulness-Based Cognitive Therapy (MBCT) (28). Mindfulness as a mediator was further substantiated by a more recent systematic review on mechanisms of change of IMIs for depression (43). The current study extends these existing findings by employing multiple assessment times and the method of time-lagged mediation analysis (71). This enabled the establishment of temporal precedence, an essential criterion for mediation (23) and a shortcoming of many preexisting studies. Finally, our results align with the theoretical assumption of mindfulness-based approaches that developing mindfulness is a key mechanism for improving mental health outcomes (16). Taken together, the promotion of mindfulness appears to be an “active ingredient” in the improvement of (subthreshold) depressive symptoms and might be routinely incorporated in mental health promotion IMIs. However, recent evidence on potential negative effects of mindfulness under certain circumstances (e.g., derealization, anxiety) should be considered (25, 83, 84).

None of the other mediator variables could be confirmed within our analyses. Alexithymia was found to significantly decrease in the intervention groups of StudiCare Mindfulness in the main analysis of this RCT (25), which is in line with previous meta-analytic evidence (42). However, we were unable to demonstrate a mediating role of this construct in the improvement of depressive symptoms. No other trial so far has studied alexithymia as a mediator of mindfulness-based interventions. As of today, it is still unclear whether alexithymia predisposes to depression, is a reaction to depression or whether these two constructs simply co-occur (85). Prospective epidemiological research and finegrained mediation studies may shed some light on these questions.

We could also not replicate previous findings of a mediating role of emotion regulation (ER) on the effectiveness of mindfulness-based IMIs by Ma and colleagues (38) and Sanilevici and colleagues (39). This might be explained by the fact that their study only employed cross-sectional mediation analysis, which does not allow for definite conclusions about the timeline of change and the establishment of mediation (71). Thus theoretically, the relationship between ER and depression could have been correlational or backwards. Additionally, both Ma and colleagues (38) and Sanilevici and colleagues (39) employed the “Difficulties in Emotion Regulation Scale” (DERS) (86) instead of the “Emotion Regulation Questionnaire” (ERQ) (36) we used. The DERS captures different facets of emotion regulation, such as “lack of emotional awareness”, “lack of emotional clarity”, or “non-acceptance of emotional responses”. These facets might be more sensitive to change through mindfulness-based interventions and therefore lead to significant effects on depressive symptoms. More research employing longitudinal mediation designs and various measures of ER are needed to unravel the potential mediating role of ER in the context of mindfulness-based IMIs.

Similarly to ER, evidence for self-efficacy (SE) is very scarce but suggests this construct as a potential mediator of IMIs' effectiveness on depressive symptoms (47). Our study was unable to replicate these findings. However, the study conducted by Clarke and colleagues (47) also only allowed for cross-sectional mediation analyses. Additionally, the construct of mental health SE examined in their study is not directly comparable to the construct of perceived SE (63) analyzed in the current trial. Just as in the case of ER, more studies employing longitudinal research designs and various instruments for measuring SE are needed to evaluate this construct's role as a potential mediator of IMI effectiveness on depression.

The non-significant findings concerning the potential mediational role of cognitive fusion (CF) are somewhat surprising, as previous meta-analytic evidence demonstrated mediation of the effectiveness of Acceptance and Commitment Therapy (ACT) (29) in the face-to-face setting by CF (22). One explanation for this discrepancy could be that StudiCare Mindfulness is not exclusively based on ACT, but rather includes elements of ACT alongside elements from MBSR (25) and general stress management techniques (59). Compared to ACT, more emphasis was placed on mindful meditation practice (every intervention module), whereas cognitive defusion was only explicitly addressed in two modules. Although cognitive fusion was significantly reduced in both intervention groups (*d* = −0.52–−0.49, 95% CI −0.77–−0.25) (25), it appears not to be a driving force of the effectiveness of StudiCare Mindfulness on depressive symptoms.

As a final remark, in the sensitivity analysis conducted within the current study, the indirect effects of both SE and CF approximated statistical significance (*p* = 0.6). This might indicate that these variables play a mediational role for the effectiveness of (mindfulness-based) IMIs that we were unable to detect in our study, e.g., due to insufficient power. Consequently, it might be worthwhile to further examine these constructs in adequately powered, meticulously planned mediation studies.

### 4.2. Moderation analysis

Concerning the moderation analyses, most of the examined variables could not be confirmed as moderators of the effectiveness of StudiCare Mindfulness on both depressive symptom severity and mindfulness. These findings are in line with a previous study examining the effectiveness of a guided version of StudiCare Mindfulness. In this study, we did not find sociodemographic variables, baseline mindfulness, baseline symptomatology, psychotherapy experience and attitudes towards IMIs to moderate the intervention's effectiveness on mindfulness (72). Somewhat surprisingly, we found the number of semesters to have a moderating role in the effectiveness of StudiCare Mindfulness on depressive symptom severity in the current trial. College students with a higher number of semesters benefited less from the intervention. As this is an exploratory finding and, to our knowledge, no other studies have explored the potential moderating effect of this variable so far, confirmative studies will have to show if these results can be validated.

Concerning the outcome mindfulness, results of this study demonstrated that the intervention's effectiveness is moderated by baseline mindfulness and baseline SE. We found lower baseline levels are associated with larger intervention effects, whereas the moderation found by Shapiro and colleagues (51) was in the other direction, i.e., higher baseline mindfulness was associated with greater improvements (51). Potential explanations are that StudiCare Mindfulness was designed for “mindfulness beginners” and lower baseline levels might leave more room for improvement.

Concerning baseline SE, we found this variable to moderate the intervention's effectiveness on mindfulness, with lower baseline SE being associated with larger intervention effectiveness on mindfulness. This aligns with existing evidence by Clark and colleagues (47), which found participants with low mental health efficacy to benefit more from an IMI for depression. Insofar, results of the moderation analyses might indicate that the intervention is particularly beneficial for college students low on mindfulness and SE. However, our findings are exploratory, existing evidence is still scarce, and more research is needed to substantiate the results of this secondary analyses.

Overall, StudiCare Mindfulness was found to effectively increase mindfulness and reduce depressive symptoms regardless of most baseline variables in two different studies (25, 72) examining different guidance formats. This suggests that the IMI might be a universally applicable intervention for improving college students' psychological well-being, which is an important prerequisite for large-scale implementation.

### 4.3. Strengths, limitations and future directions

The presented mediation study has several strengths. First, the study is of high methodological quality, featuring elements such as a randomized controlled design, theoretically derived mediator variables, multiple assessment time points (with one assessment occurring during the intervention), and the employment of a rigorous statistical method of analyses, i.e., time-lagged mediation analysis (71), which enabled the establishment of temporal precedence (23). Consequently, many of the criteria Kadzin (23) proposed to establish mediation are met. Further, the current study examined potential mediator variables of mindfulness-based IMI derived from theory that have not been investigated systematically so far, i.e., SE, ER, and alexithymia.

However, there are also multiple limitations and directions for future research. First, the sample size was larger than in many other mediation studies in the field of mindfulness-based IMIs (21). Nonetheless, moderation and mediation analyses might still have been underpowered, as such analyses generally necessitate very large sample sizes (87). A related matter concerns that this RCT was designed and powered to evaluate the effectiveness of the primary outcome mindfulness rather than to identify mechanisms of change. Although we did consider secondary mediation analyses in the trial design *a priori* (26), future confirmatory mediation studies should take further aspects of rigorous meditation study planning into account (23), such as more than one assessment point during the intervention, in the best case after every module. This would enable a more detailed mapping of change processes, e.g., the identification of mechanisms of change that are relevant for specific intervention modules.

Further, it cannot be ruled out that a third variable not assessed within our study design caused the change in both the outcome and the mediator (88). Although this possibility can never be ruled out completely, the evidence for the causal role of a mediator such as mindfulness might be further strengthened by experimental manipulation. For example, a future RCT could compare two intervention versions with different doses of mindfulness exercises. Another limitation relates to the very low utilization of guidance in the GoD group, thus both groups (unguided, “guidance on demand”) were combined into one intervention group. As a consequence, we were unable to examine mediator and moderator variables that might be unique to these specific guidance formats. Future trials comparing full guidance to no guidance could examine such potentially unique effects or even guidance itself as a mechanism of change (43).

Concerning the moderation analyses, due to the investigated sample's nature, there was a range restriction for several potential moderator variables, which limits the generalizability of results. For example, this regards age (college students), gender (primarily female participants), baseline mindfulness (high mindfulness was an exclusion criterium), and credibility/expectancy (positive due to self-selection, no incentives). Non-significant moderator variables might still be significant if examined in samples which are not subject to the mentioned range restrictions. Another limitation is that we employed a waitlist control group instead of an active control group, which does not allow for the examination of the specificity of mediators (23). Future studies could investigate the specificity of mediators of mindfulness-based IMIs by choosing active control conditions such as Cognitive Behavioral Therapy. This would allow conclusions about whether mindfulness is indeed specific to MBSR/ACT rather than a common mechanism of change of psychotherapy.

Finally, our analyses only included participants who received a minimal intervention dose (i.e., one module). This decision was made to increase the probability of detecting existing mediators or moderators within the exploratory context of this study. However, this approach might also have introduced bias, as completely non-adherent participants were excluded (23). Future studies could examine the influence of intervention adherence on the mediating effect of different variables via moderated mediation analyses.

## 5. Conclusions

In conclusion, the results of this study indicate that mindfulness is a causal mediator of the effectiveness of the Internet- and mobile-based intervention (IMI) StudiCare Mindfulness on depressive symptom severity. In addition, moderation analyses demonstrated that the intervention's effectiveness is independent of most baseline characteristics, suggesting the effectiveness of the intervention in college students with a wide array of baseline characteristics. These findings support mindfulness' beneficial role in improving psychological health, the capacity of IMIs to successfully teach these skills and the universal applicability of mindfulness-based IMI for college student mental health promotion. Future confirmative studies will have to substantiate these findings.

## Data Availability

The data analyzed in this study is subject to the following licenses/restrictions: External researches may get access to the final trial dataset from HB on request, depending on to be specified data security and data exchange regulation agreements. To ensure confidentiality, data dispersed to any investigator or researcher will be blinded of any identifying participant information. Requests to access these datasets should be directed to harald.baumeister@uni-ulm.de.
