# Supplementary material for: Moderators and mediators of change of an internet-based mindfulness intervention for college students: secondary analysis from a randomized controlled trial
Source: Front Digit Health. 2023 Jun 27;5:1179216. doi: 10.3389/fdgth.2023.1179216 (PMC10333756; doi:10.3389/fdgth.2023.1179216)
Supplement: Supplementary file 1 [file Datasheet1.docx]

Supplementary Material

**Moderators and mediators of change of an Internet-based mindfulness intervention for college students: Secondary analyses from a randomized controlled trial**

**Ann-Marie Küchler*, Fanny Kählke, Leandra Bantleon, Yannik Terhorst, David Daniel Ebert, Harald Baumeister**

*** Correspondence:** Ann-Marie Küchler: ann-marie.kuechler@uni-ulm.de

# Supplementary Figures

***
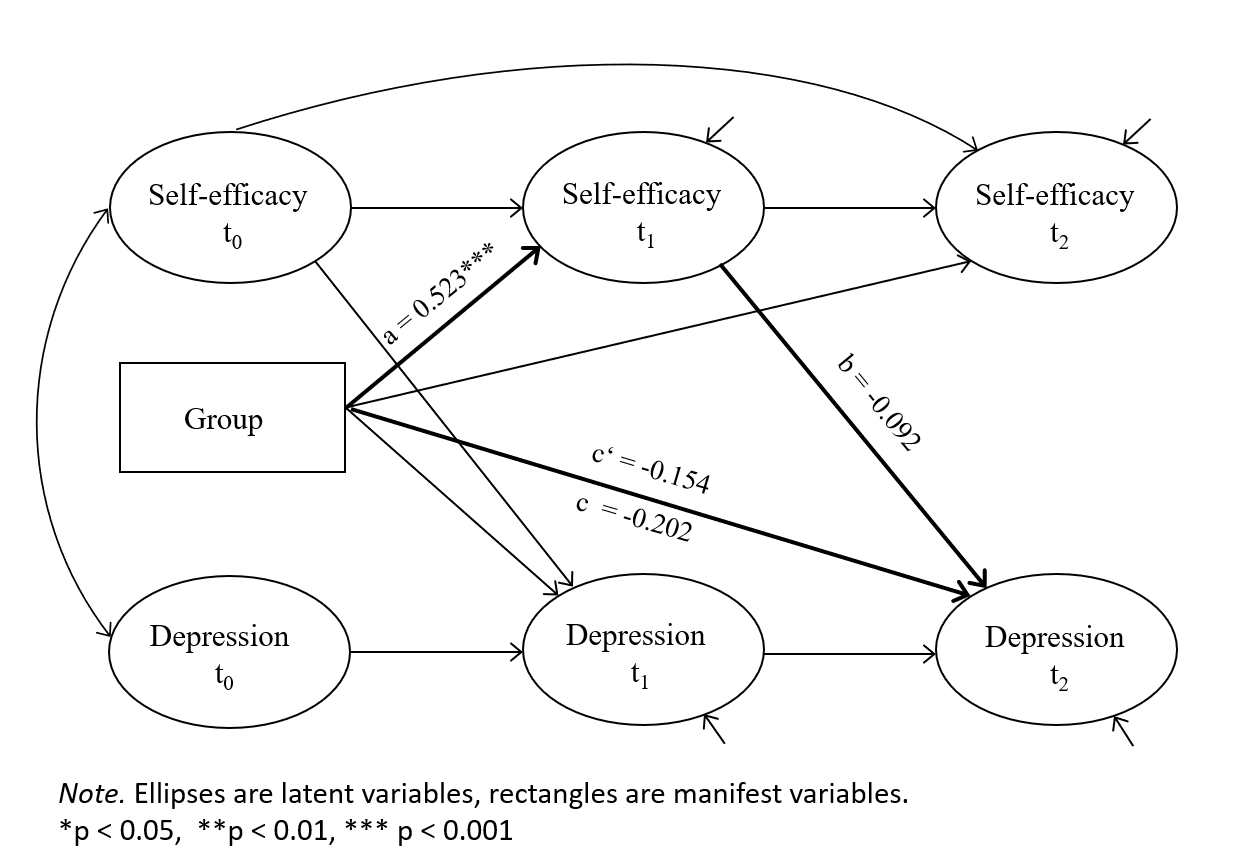
***

**Supplementary Figure 1.** Path model for the mediator cognitive reappraisal.

*Note.* Ellipses are latent variables, rectangles are manifest variables. *****p < 0.005, ** p < 0.01, *** p < 0.001

***
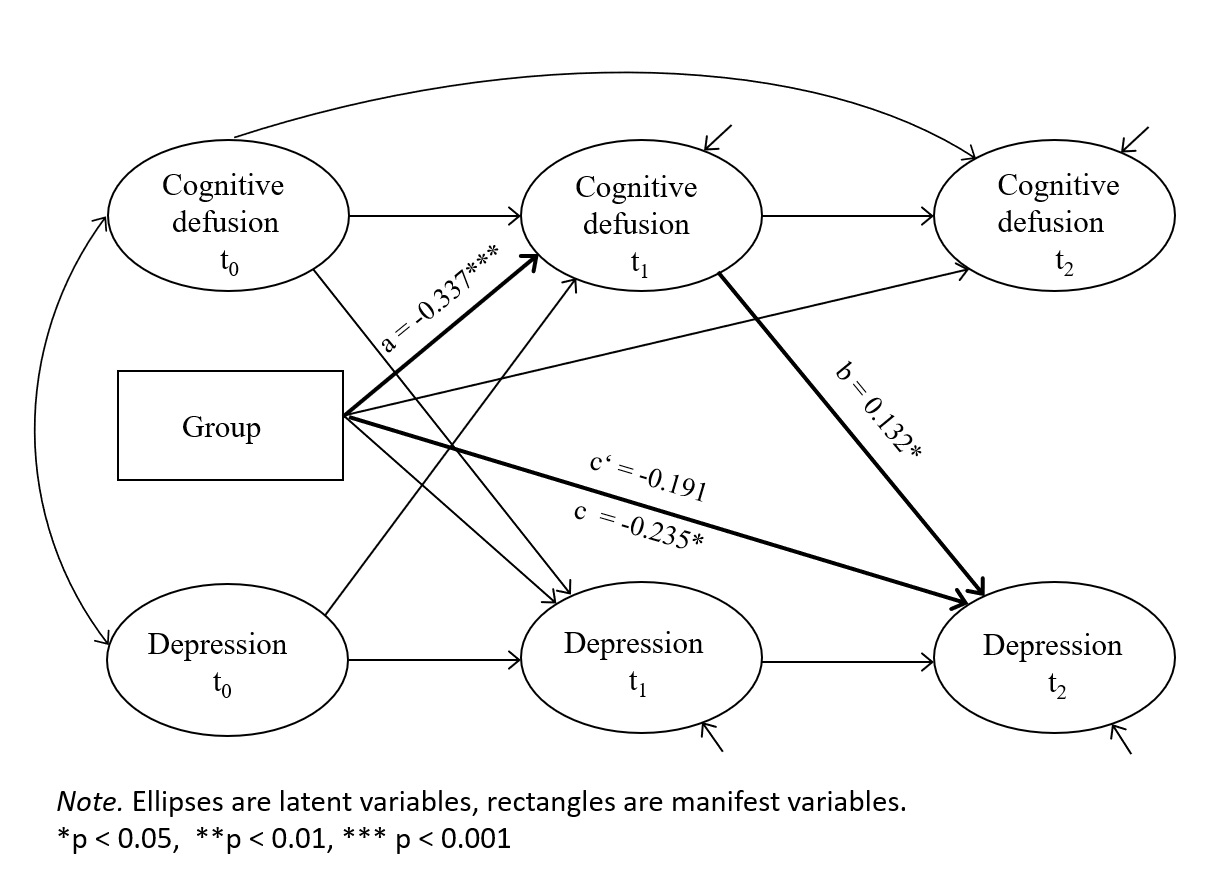
***

**Supplementary Figure 2.** Path model for the mediator cognitive defusion.

*Note.* Ellipses are latent variables, rectangles are manifest variables. *****p < 0.005, ** p < 0.01, *** p < 0.001

***
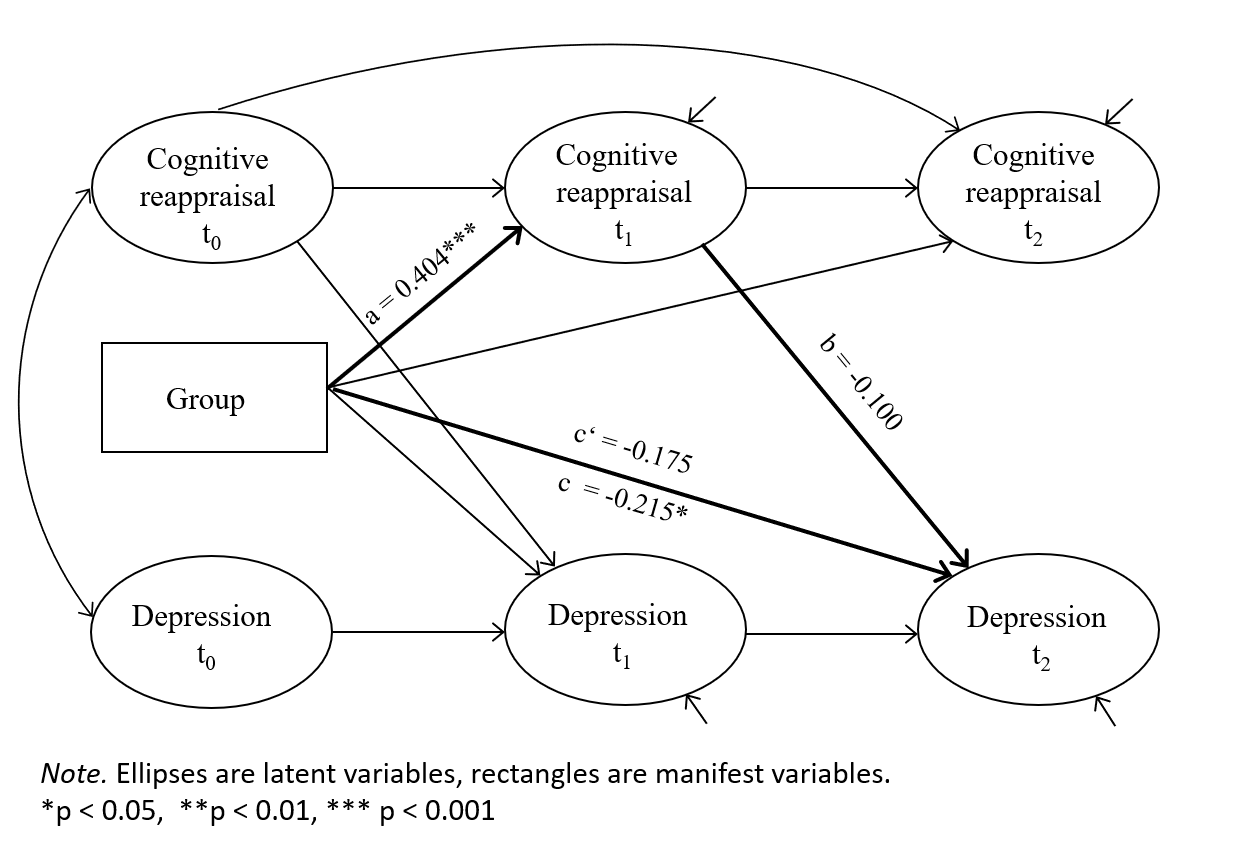
***

**Supplementary Figure 3.** Path model for the mediator cognitive reappraisal.

*Note.* Ellipses are latent variables, rectangles are manifest variables. *****p < 0.005, ** p < 0.01, *** p < 0.001

***
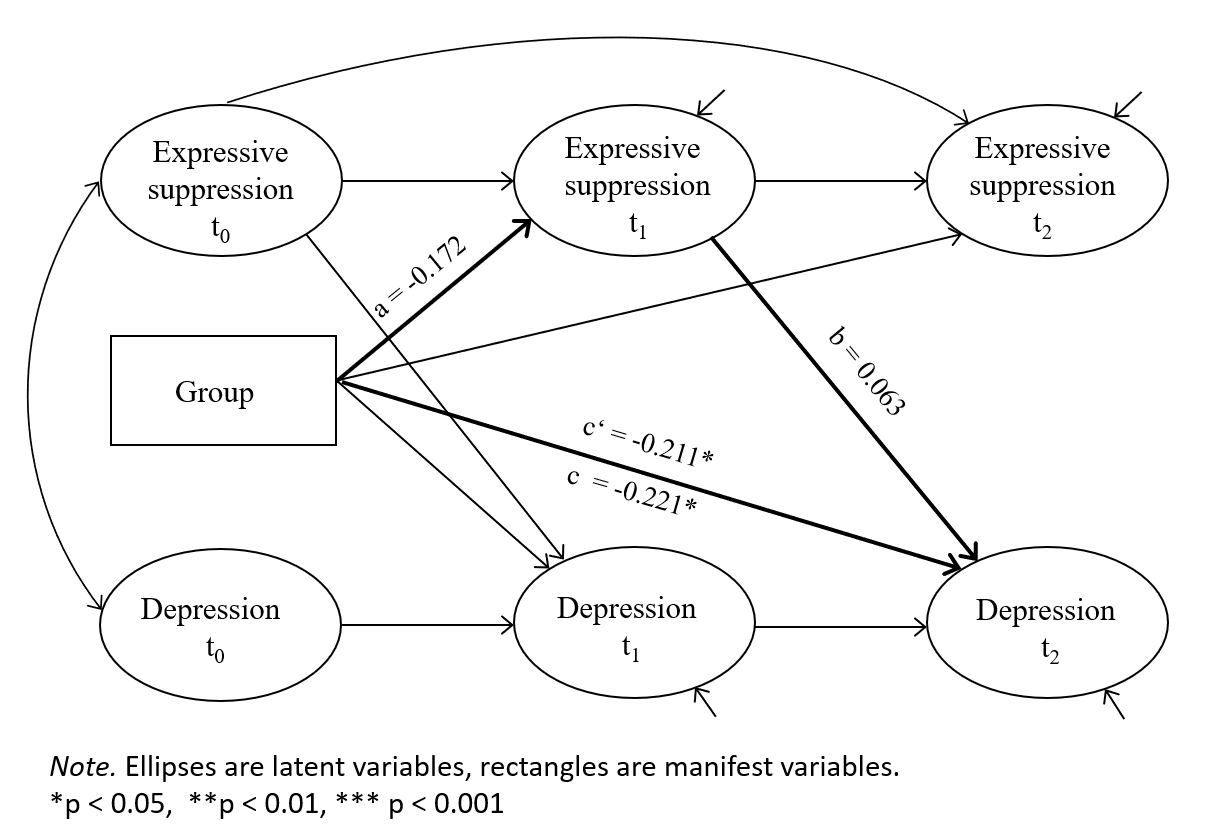
***

**Supplementary Figure 4.** Path model for the mediator expressive suppression.

*Note.* Ellipses are latent variables, rectangles are manifest variables. *****p < 0.005, ** p < 0.01, *** p < 0.001

***
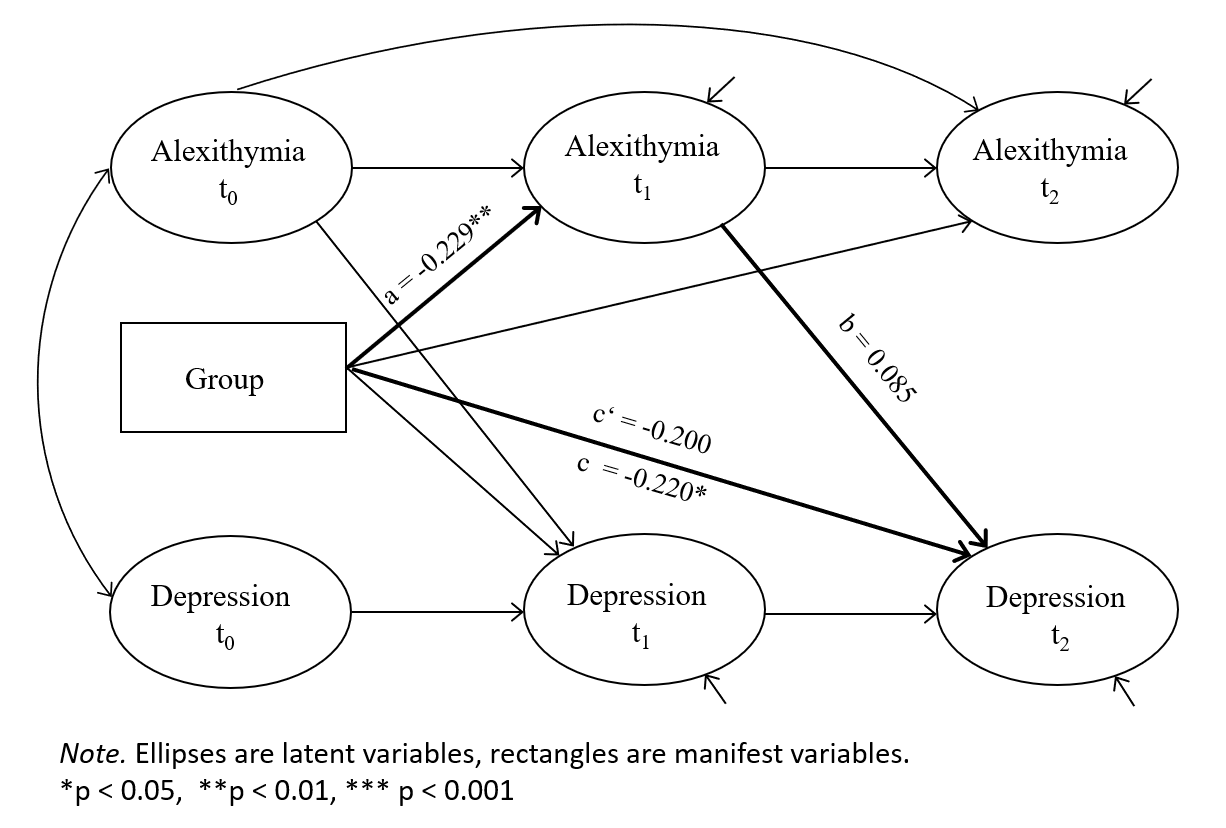
***

**Supplementary Figure 5.** Path model for the mediator alexithymia.

*Note.* Ellipses are latent variables, rectangles are manifest variables. *****p < 0.005, ** p < 0.01, *** p < 0.001

# Supplementary Tables

**Supplementary Table 1.** Results of a longitudinal mediation analysis with three measurement time points, excluding the direct effect (c-path)

| Mediator | a-path | b-path | Indirect effect  a x b | RMSEA | SRMR |
| --- | --- | --- | --- | --- | --- |
| Mindfulness | 0.834*** | -0.195** | -0.163** | 0.042 | 0.077 |
| Self-efficacy | 0.526*** | -0.119* | -0.063 | 0.041 | 0.074 |
| Cognitive fusion | -0.341*** | 0.142* | -0.048 | 0.042 | 0.066 |
| ER– CR | 0.409*** | -0.118* | -0.048 | 0.044 | 0.071 |
| Alexithymia | -0.231** | 0.093 | -0.021 | 0.046 | 0.083 |

*Note.* The outcome was PHQ-9 depression symptom severity for each mediator. Coefficients are standardized. ER-CR = cognitive reappraisal. ER-ES = expressive suppression.

* p < 0.05. ** p < 0.01. *** p < 0.001.

Supplementary Table 2. Moderation analyses for the dependent variable depressive symptom severity at t2

|  | **Beta** | ***SE*** | ***t*** | **95% CI** | ***p*** |
| --- | --- | --- | --- | --- | --- |
| Baseline Mindfulness (FMI) | | | | | |
| Intercept | 0.25 | 0.09 | 2.70 | 0.07; 0.43 | 0.007 |
| Group | -0.40 | 0.12 | -3.25 | -0.64; -0.16 | 0.001 |
| Moderator | -0.29 | 0.10 | -2.89 | -0.49; -0.09 | 0.005 |
| Group x Moderator | 0.06 | 0.12 | 0.49 | -0.17; 0.28 | 0.628 |
|  |  |  |  |  |  |
| Baseline Depression (PHQ-9) | | | | | |
| Intercept | 0.27 | 0.08 | 3.19 | 0.10; 0.44 | 0.002 |
| Group | -0.43 | 0.11 | -3.78 | -0.65; -0.20 | <0.001 |
| Moderator | 0.57 | 0.09 | 6.52 | 0.40; 0.74 | <0.001 |
| Group x Moderator | -0.14 | 0.11 | -1.27 | -0.35; 0.07 | 0.205 |
|  |  |  |  |  |  |
| Baseline Self-Efficacy (SES) | | | | | |
| Intercept | 0.24 | 0.09 | 2.63 | 0.06; 0.43 | 0.009 |
| Group | -0.39 | 0.12 | -3.17 | -0.64; -0.15 | <0.001 |
| Moderator | -0.20 | 0.10 | -1.97 | -0.40; 0.00 | 0.051 |
| Group x Moderator | 0.01 | 0.12 | 0.06 | -0.23; 0.24 | 0.953 |
|  |  |  |  |  |  |
| Credibility/Expectancy (CEQ) | | | | | |
| Intercept | 0.26 | 0.09 | 2.70 | 0.07; 0.44 | 0.007 |
| Group | -0.40 | 0.12 | -3.25 | -0.65; -0.16 | 0.001 |
| Moderator | -0.11 | 0.10 | -1.13 | -0.31; 0.08 | 0.259 |
| Group x Moderator | -0.07 | 0.12 | -0.57 | -0.30; 0.17 | 0.570 |
|  |  |  |  |  |  |
| Age | | | | | |
| Intercept | 0.25 | 0.09 | 2.68 | 0.07; 0.44 | 0.008 |
| Group | -0.41 | 0.13 | -3.23 | -0.65; -0.16 | 0.002 |
| Moderator | 0.00 | 0.09 | 0.00 | -0.18; 0.18 | 0.999 |
| Group x Moderator | 0.01 | 0.12 | 0.08 | -0.22; 0.24 | 0.940 |
|  |  |  |  |  |  |
| Gender | | | | | |
| Intercept | 0.29 | 0.21 | 1.41 | -0.12; 0.71 | 0.119 |
| Group | -0.24 | 0.25 | -0.96 | -0.73; 0.25 | 0.336 |
| Moderator | -0.05 | 0.25 | -0.21 | -0.54; 0.44 | 0.832 |
| Group x Moderator | -0.22 | 0.29 | -0.75 | -0.80; 0.36 | 0.451 |
|  |  |  |  |  |  |
| Nationality | | | | | |
| Intercept | 0.48 | 0.24 | 2.03 | 0.01; 0.95 | 0.044 |
| Group | -0.74 | 0.28 | -2.67 | -1.28; -0.19 | 0.008 |
| Moderator | -0.28 | 0.27 | -1.02 | -0.81; 0.26 | 0.311 |
| Group x Moderator | -0.41 | 0.31 | 1.30 | -0.21; 1.03 | 0.196 |
|  |  |  |  |  |  |
| Number of semesters | | | | | |
| Intercept | 0.26 | 0.09 | 2.73 | 0.07; 0.45 | 0.007 |
| Group | -0.41 | 0.13 | -3.25 | -0.65; -0.16 | 0.001 |
| Moderator | -0.14 | 0.08 | -1.61 | -0.30; 0.03 | 0.108 |
| Group x Moderator | 0.24 | 0.11 | 2.12 | 0.02; 0.47 | **0.035** |
|  |  |  |  |  |  |
| Marital Status | | | | | |
| Intercept | 0.28 | 0.12 | 2.34 | 0.04; 0.51 | 0.020 |
| Group | -0.39 | 0.15 | -2.60 | -0.69; -0.10 | 0.010 |
| Moderator | -0.06 | 0.20 | -0.30 | -0.45; 0.33 | 0.762 |
| Group x Moderator | -0.05 | 0.25 | -0.19 | -0.53; 0.44 | 0.847 |
|  |  |  |  |  |  |
| Psychotherapy Experience | | | | | |
| Intercept | 0.15 | 0.12 | 1.33 | -0.07; 0.38 | 0.184 |
| Group | -0.33 | 0.14 | -2.29 | -0.61; -0.04 | 0.023 |
| Moderator | 0.34 | 0.23 | 1.56 | -0.09; 0.76 | 0.120 |
| Group x Moderator | -0.22 | 0.28 | -0.76 | -0.76; 0.35 | 0.450 |
|  |  |  |  |  |  |
| Mindfulness Experience | | | | | |
| Intercept | 0.33 | 0.11 | 3.06 | 0.12; 0.55 | 0.002 |
| Group | -0.37 | 0.14 | -2.59 | -0.65; -0.09 | 0.010 |
| Moderator | -0.24 | 0.20 | -1.20 | -0.63; 0.15 | 0.231 |
| Group x Moderator | -0.05 | 0.26 | -0.18 | -0.56; 0.47 | 0.860 |
|  |  |  |  |  |  |
| Alternative support offers | | | | | |
| Intercept | 0.11 | 0.13 | 0.89 | -0.13; 0.35 | 0.373 |
| Group | -0.32 | 0.15 | -2.06 | -0.62; -0.01 | 0.042 |
| Moderator | 0.45 | 0.21 | 2.14 | 0.03; 0.87 | 0.034 |
| Group x Moderator | -0.25 | 0.27 | -0.94 | -0.77; 0.28 | 0.350 |
| *Note.* *M* mean; *SD* standard deviation; *CI* Confidence interval; CEQ Credibility-/Expectancy Questionnaire; FMI Freiburg Mindfulness Inventory; PHQ-9 Patient Health Questionnaire-9; SES Self-Efficacy Scale.  Significant interactions are printed bold. | | | | | |

Supplementary Table 3. Moderation analyses for the dependent variable mindfulness at t2

|  | **Beta** | ***SE*** | ***t*** | **95% CI** | ***p*** |
| --- | --- | --- | --- | --- | --- |
| Baseline Mindfulness (FMI) | | | | | |
| Intercept | -0.60 | 0.07 | -7.72 | -0.75; -0.44 | <0.001 |
| Group | 0.95 | 0.10 | 9.23 | 0.75; 1.15 | <0.001 |
| Moderator | 0.54 | 0.08 | 7.12 | 0.39; 0.69 | <0.001 |
| Group x Moderator | -0.20 | 0.10 | -2.00 | -0.41; -0.00 | **0.047** |
|  |  |  |  |  |  |
| Baseline Depression (PHQ-9) | | | | | |
| Intercept | -0.61 | 0.09 | -7.09 | -0.78; -0.44 | <0.001 |
| Group | 0.97 | 0.11 | 8.53 | 0.74; 1.19 | <0.001 |
| Moderator | -0.08 | 0.09 | -0.88 | -0.27; 0.10 | 0.383 |
| Group x Moderator | 0.05 | 0.11 | 0.48 | -0.17; 0.28 | 0.633 |
|  |  |  |  |  |  |
| Baseline Self-Efficacy (SES) | | | | | |
| Intercept | -0.59 | 0.08 | -7.29 | -0.75; -0.43 | <0.001 |
| Group | 0.94 | 0.11 | 8.77 | 0.73; 1.15 | <0.001 |
| Moderator | 0.48 | 0.08 | 5.93 | 0.32; 0.64 | <0.001 |
| Group x Moderator | -0.27 | 0.11 | -2.53 | -0.48; -0.06 | **0.012** |
|  |  |  |  |  |  |
| Credibility/Expectancy (CEQ) | | | | | |
| Intercept | -0.61 | 0.08 | -7.17 | -0.77; -0.44 | <0.001 |
| Group | 0.96 | 0.11 | 8.62 | 0.74; 1.18 | <0.001 |
| Moderator | 0.09 | 0.09 | 1.02 | -0.08; 0.26 | 0.309 |
| Group x Moderator | 0.11 | 0.11 | 1.00 | -0.10; 0.32 | 0.320 |
|  |  |  |  |  |  |
| Age | | | | | |
| Intercept | -0.60 | 0.08 | -7.04 | -0.77; -0.43 | <0.001 |
| Group | 0.95 | 0.11 | 8.50 | 0.73; 1.17 | <0.001 |
| Moderator | -0.22 | 0.08 | -2.72 | 0.37; -0.06 | 0.007 |
| Group x Moderator | 0.12 | 0.10 | 1.20 | -0.08; 0.33 | 0.231 |
|  |  |  |  |  |  |
| Gender | | | | | |
| Intercept | -0.49 | 0.18 | -2.67 | -0.86; -0.13 | 0.009 |
| Group | 0.80 | 0.23 | 3.51 | 0.35; 1.26 | <0.001 |
| Moderator | -0.15 | 0.21 | -0.73 | -0.57; 0.26 | 0.464 |
| Group x Moderator | 0.21 | 0.25 | 0.84 | -0.29; 0.71 | 0.405 |
|  |  |  |  |  |  |
| Nationality | | | | | |
| Intercept | -0.87 | 0.21 | -4.19 | -1.28; -0.46 | <0.001 |
| Group | 1.33 | 0.24 | 5.47 | 0.85; 1.81 | <0.001 |
| Moderator | 0.31 | 0.23 | 1.36 | -0.14; 0.77 | 0.175 |
| Group x Moderator | -0.46 | 0.28 | -1.64 | -1.01; 0.09 | 0.103 |
|  |  |  |  |  |  |
| Number of semesters* | | | | | |
| Intercept | -0.60 | 0.08 | -7.10 | -0.77; -0.44 | <0.001 |
| Group | 0.96 | 0.11 | 8.55 | 0.74; 1.18 | <0.001 |
| Moderator | -0.16 | 0.08 | -1.94 | -0.32; 0.00 | 0.053 |
| Group x Moderator | 0.04 | 0.11 | 0.38 | -0.17; 0.25 | 0.703 |
|  |  |  |  |  |  |
| Marital Status | | | | | |
| Intercept | -0.53 | 0.11 | -4.89 | -0.74; -0.32 | <0.001 |
| Group | 0.89 | 0.14 | 6.48 | 0.62; 1.15 | <0.001 |
| Moderator | -0.21 | 0.17 | -1.23 | -0.55; 0.13 | 0.220 |
| Group x Moderator | 0.20 | 0.22 | 0.93 | -0.23; 0.63 | 0.353 |
|  |  |  |  |  |  |
| Psychotherapy Experience | | | | | |
| Intercept | -0.54 | 0.10 | -5.37 | -0.74; -0.34 | <0.001 |
| Group | 0.91 | 0.13 | 7.11 | 0.66; 1.17 | <0.001 |
| Moderator | -0.22 | 0.19 | -1.18 | -0.59; 0.15 | 0.240 |
| Group x Moderator | 0.13 | 0.25 | 0.54 | -0.36; 0.62 | 0.590 |
|  |  |  |  |  |  |
| Mindfulness Experience | | | | | |
| Intercept | -0.81 | 0.10 | -7.99 | -1.01; -0.61 | <0.001 |
| Group | 1.04 | 0.14 | 7.66 | 0.77; 1.30 | <0.001 |
| Moderator | 0.61 | 0.18 | 3.36 | 0.25; 0.97 | <0.001 |
| Group x Moderator | -0.28 | 0.23 | -1.19 | -0.74; 0.18 | 0.234 |
|  |  |  |  |  |  |
| Alternative support offers | | | | | |
| Intercept | -0.54 | 0.10 | -5.25 | -0.75; -0.34 | <0.001 |
| Group | 0.99 | 0.13 | 7.67 | 0.73; 1.24 | <0.001 |
| Moderator | -0.20 | 0.20 | -1.00 | -0.58; 0.19 | 0.319 |
| Group x Moderator | -0.10 | 0.24 | -0.40 | -0.57; 0.38 | 0.689 |
| *Note.* *M* mean; *SD* standard deviation; *CI* Confidence interval; CEQ Credibility-/Expectancy Questionnaire; FMI Freiburg Mindfulness Inventory; PHQ-9 Patient Health Questionnaire-9; SES Self-Efficacy Scale.  Significant interaction effects are bold.  *outliers removed | | | | | |
